# Socioeconomic inequality in Latin America and the Caribbean: the post-pandemic future for the training of health professionals

**DOI:** 10.1590/S0104-59702023000100029

**Published:** 2023-08-14

**Authors:** Lina Faria, Rocío Elizabeth Chavez Alvarez, Luiz Antonio de Castro Santos

**Affiliations:** i Professora associada e coordenadora institucional, Mestrado Profissional em Saúde da Família/Universidade Federal do Sul da Bahia.Porto Seguro – BA – Brasil lina@ufsb.edu.br; ii Professora permanente, Mestrado Profissional em Saúde da Família/ Universidade Federal do Sul da Bahia.Porto Seguro – BA – Brasil chioliz2014@gmail.com; iii Docente permanente (colaborador), Mestrado Profissional em Saúde da Família/Universidade Federal do Sul da Bahia.Porto Seguro – BA – Brasil lacs1945@gmail.com

**Keywords:** Social inequality, Health inequity, Pandemic, Educational practices, Social protection, Desigualdades sociais, Iniquidade na saúde, Pandemia, Práticas educativas, Proteção social

## Abstract

Inequality is a global, structural problem that is particularly marked in the world’s poorest countries. The covid-19 pandemic exacerbated this historic problem in Latin America and the Caribbean and deepened uncertainties in relation to basic human needs. This study presents an overview of the subject on the basis of official reports from international agencies (PAHO, WHO, ECLAC) between 2019 and 2022 and discusses some paths for the training of health professionals in Brazil. It also investigates how health practices could be changed to ensure greater social protection for vulnerable populations, based on the proposals of Paulo Freire and Edgar Morin, which highlight current social and health problems.

## Resumo

A desigualdade é um problema global e estrutural que aflige com maior intensidade as populações dos países mais empobrecidos. A pandemia da covid-19 agravou esse problema histórico na América Latina e Caribe e aprofundou as incertezas relacionadas às necessidades humanas básicas. O estudo apresenta um painel sobre o tema produzido pelos relatórios oficiais de agências internacionais (Opas, OMS, Cepal) entre 2019 e 2022, e discute alguns caminhos para a formação profissional em saúde no Brasil, bem como as mudanças nas práticas em saúde que podem impulsionar a proteção social das populações vulneráveis, com base nas propostas de Paulo Freire e Edgar Morin, que colocam em evidência as problemáticas sociossanitárias atuais.

The covid-19 pandemic has exacerbated historic, structural inequalities in Latin America and the Caribbean and brought about increased uncertainty in regard to basic human needs. The social and health crisis has been a factor in producing greater inequality, resulting in new social and economic situations. This inequality is often reflected in the field of health, especially when it comes to access to health services ( Barreto, 2017 ; OECD, 2020 ; Faria, Patiño, 2020). Latin America and the Caribbean is one of the regions that has been most affected by the pandemic, in both numbers of cases and numbers of deaths. Although it is home to just over 8% of the world’s population, by December 2020 it accounted for around 20% of cases and more than 25% of deaths worldwide (Montes, Fariza, 4 mar. 2021; Cepal, 2021a). By October 2021, 12% of all covid-19-related deaths were in Brazil, even though more than 80% of the Brazilian population was fully vaccinated ( Our World in Data, 2021 , 2022 ; Brasil, 2021 , 2022 ).^1^

It is fair to say that the immediate legacy of the pandemic is a more unequal world. Eurocentric models of social protection based on the precepts of liberalism and meritocracy have failed to resolve the social inequalities in Latin America and the Caribbean ( Basile, 2020 , p.3559). The disparity between rich and poor grew significantly between 2019 and 2022, accentuated by privatization policies and the erosion of social rights. Brazil ranks among the ten most unequal countries in the world: the richest 10% of its population earn more than ten times the minimum wage and concentrate 59% of the total national income, while more than half of the population collectively earn about 10% of total national income (Pellicer, Grasso, 7 dez. 2021).

A more unequal world is also the legacy of capitalist societies. According to Rehbein (2020 , p.696) “the ideological view of capitalism combines with the invisibility of structural domination to construct an entirely misguided understanding of inequality.” For the author, capitalist thinking should be centered on the idea of competition on equal terms for a better quality of life. Instead, inequality is more evident than ever in contemporary societies, and the health crisis caused by the pandemic has only highlighted these gaping inequalities, as Sousa Santos (2 abr. 2020) calls them, as well as the need for new public policies and new social compacts. For Sousa Santos (2 abr. 2020, p.1), “as neoliberalism has taken over as the dominant version of capitalism and capitalism has become increasingly subservient to the logic of the financial sector, the world has started to live in a permanent state of crisis.”

The first part of the study presents an overview based on official documents published by the Economic Commission for Latin America and the Caribbean (ECLAC), the Pan American Health Organization, and the World Health Organization (WHO) for 2019-2022 as well as other documents from the same period used in the international course “La pandemia y la salud pública en latinoamérica” [The pandemic and public health in Latin America].^2^ The documentation reveals a complex scenario whose most critical elements still need to be understood, taking a multidimensional, holistic view of the social phenomena in question, with emphasis on the complex and precarious nature of the health, economic, political, and social structures that have given rise to these phenomena in so many Latin American and Caribbean countries.

It is important to note that several reports published by these agencies indicate that one way to transform reality (fighting poverty and tackling socioeconomic inequalities) is to invest in social protection policies geared towards the most vulnerable populations, such as investments in the care economy,^3^ which have the effect of mitigating the damage caused by social inequality, while also promoting inclusive and sustainable economic growth and reducing violence and social injustice ( UN Women, 2020 ).

It could be said that social protection constitutes a “gateway” for social policies, providing investments in basic needs to meet societies’ most pressing demands. Therefore, there is an urgent need to fight inequality, whether it be related to skin color, race, gender, health, economic, social, or politics, by identifying and executing targeted actions ( Opas/OMS, 2012 , 2017 , 2019 , 2020 ). However, what has been observed since the beginning of the pandemic in Latin America and the Caribbean has been

an indefinite epidemiological threshold of rising case numbers for the region, together with an ascending curve of collective panic, misinformation/overinformation, a naturalization of lack of protections in exclusionary societies, a radicalization of gender, ethnic, and social inequality, a certain invisibility of the structural weaknesses of public health systems, and decision-making based on the shock doctrine applied to society ( Basile, 2020 , p.3558).

Investment in the care economy should therefore consider the immediate impacts of the pandemic on health systems, particularly gender inequality. Before covid-19, health services were already stretched in several Latin American and Caribbean countries, but the pandemic pushed them to breaking point. This is why it is so important for investments to be made in social protection, including health services and policies to enhance the value of health workers, which implies the need for long-term education and health policies to meet the needs of the public health system (Sistema Único de Saúde, SUS).

For some time, ECLAC publications have contained discussions of the importance of the care economy and social protection, especially in relation to gender inequality, raising important issues for debate. These include the benefits of promoting the care economy as a driver of recovery for societies in the post-pandemic context, such as efforts to eliminate structural gaps of inequality ( Cepal, 2020 , 2021a, 2021b , 2021c , 2021d , 2021e ).

In the second part – a point of inflexion in the debate – some ideas for the future of health education are discussed, with a focus on Brazil. What are the gaps and how can they be filled to reorient training in such a way as to meet the growing demands on SUS and the communities and professionals engaged in primary care? How can changes in health education be harnessed to foster the social protection of the most vulnerable populations? And how can training models contextualized in their societal realities contribute to formulations that encourage new forms of organization and challenge contradictions and social inequalities?

It is important to point out that social inequality was on the political agenda as far back as the 1970s, in the movement for a reform of the health system in Brazil. The proposals were not limited to the creation of a public health system (today’s SUS), but incorporated a broader conception of health with emphasis on its social determinants and proposed changing the health status of the Brazilian population by reducing social inequalities and improving the living conditions of different groups ( Almeida-Filho, 2011 ).

The aim here is therefore to discuss aspects of education and professional training beyond the provision of care in the field of health. The transformations seen in Brazilian society in recent years have exacerbated social inequalities so greatly that the interrelations between living conditions, social determinants of health, and professional training have become more complex (Santos, Araújo, Joazeiro, 2019). These factors mark the relationship between educational institutions and health services in the construction of knowledge necessary for education with social responsibility, as the Brazilian educator and philosopher Paulo Freire (1987 , 1992 ) and the French sociologist and philosopher Edgar Morin (2002) would say.

According to Ceccim and Feuerwerker (2004) , the task of diagnosing reality and producing meanings in the sphere of health falls to both SUS and educational institutions. As they put it, “education for the area of health should aim to transform professional practices [with social orientation] and the very way work is organized, and should be structured on the basis of a problematization of the work process and its ability to address and treat the various health-related dimensions and needs of individuals, groups, and populations” (p.43).

For Feitosa, Lago, and Feitosa (2017), for the connection between theory and practice to be strengthened, incentives must be provided for professional training both in educational institutions and in health services, since any commitment to the social reality should involve an ongoing quest for information from this reality, (re)constructing meanings and practices with a social orientation.

If we start by looking at the proposed connections between socioeconomic inequalities accentuated by the covid-19 pandemic and the training of professionals in the health service, the first thing that becomes clear is that the vulnerabilities associated with intersections of color, race, gender, and class are materialized in poor populations, and the harm caused by these phenomena does not always reach the attention of primary health care services. Nevertheless, primary care is a pivotal actor when it comes to collective, integrated, and intersectoral actions to address and prevent these phenomena, which is why it is so important for training to be based on innovative proposals for social transformation – such as those devised by Paulo Freire (1987 , 1992 ) and Edgar Morin (2002) – developing knowledge for the purposes of achieving transformation through education, which means training that can be spread throughout the country and spark in-depth debate on how to strengthen education in the SUS in dialogue with academia.

In this part, these proposals will be discussed in a bid to find areas of convergence between the thinking of Freire and Morin. It is important to note that both theorists distance themselves from the traditional pedagogical paradigm and stress the importance of building a kind of knowledge in training institutions that is based on historical and social contexts, the complexity and diversity of human beings, and the demands and needs of society “for a real transformation of reality so that, by humanizing it, men will be humanized” (Freire, 1987, p.115).

SUS, with its chronic funding shortages and difficulties in providing quality health care and access to services, is the setting where the challenges of rendering changes to health education, not to mention tackling and controlling the covid-19 pandemic, are played out in Brazil, since SUS is an umbrella for a multitude of services. As such, it offers a prime scenario for critical reflection on professional practice. Its innovations influence concepts, parameters, and principles associated with the operation of all health services. The collective problematization of experiences can trigger learning capable of transforming and overcoming conservative practices to meet the needs of society and overcome the new impasses in the health sector.

## The issue of social inequality in Latin America, with a focus on health

In Latin America, public agendas have ranged through everything from rebellion to systems of repression, from democratic stability to authoritarianism, from inclusive citizenship to exclusionary reforms. Barata (2009) has noted some aspects of this, demonstrating how inequalities reflect situations of injustice and social suffering and produce scenarios propitious for health inequity. When there are multiple inequalities, certain individuals and/or groups find themselves at a disadvantage with regard to various opportunities. In the absence of targeted public policies, this results in deteriorated, unfair, and inhumane living conditions.

There are enduring social problems in many countries, which often correlate with health inequalities across countries, regions, and social groups. The health crisis triggered by the covid-19 pandemic has amplified and exacerbated social, educational, and economic disparities, and inequality has expanded as the deficits of public health systems have grown. Health problems or diseases that should have been eradicated or controlled continue to thrive, while new covid-related health problems (sequelae) have emerged ( Opas/OMS, 2020 , 2021 ).

According to ECLAC reports, Latin America and the Caribbean is experiencing a sharp decline in living conditions, which is reflected in increased unemployment, poverty, disease, violence, and inequality. Structural inequalities also persist, especially in access to health, in a context in which the pandemic has not yet been fully controlled (Cepal, 2021a, 2021b, 2021c, 2021d, 2021e).^4^

For Cardoso (20 jan. 2021), inequalities in the region are not limited to income, but also derive from the fragmented state of the health systems, which makes access difficult, reduces coverage, and reproduces the same patterns of inequality. Public health systems coexist in tension with private health systems and their financial interests, which is not consistent with the state’s duty to provide for the population’s universal right to health, making way, instead, for a field propitious for health inequalities such as those found across the region. We are talking about systems that are outdated and have not kept pace with demographic, political, social, cultural, and scientific changes based on scientific evidence, calling for urgent transformation and retraining to meet the challenges of the 21st century.

Cotlear et al. (2015) argue that the segregation of society in terms of health is a reflection of the growing segmentation of health systems along the fault lines of economic inequality in Latin America, hampering access to social rights and greater equity. Many are the divisions that gradually morph into inequalities and often iniquities, insofar as access to and ownership of goods and services is distributed unequally according, essentially, to power relations.

In the words of Morin (9 jun. 2020), “the health crisis triggered a whole chain of interconnected crises. This polycrisis, or megacrisis, extends from the existential to the political, passing through the economy, from the individual to the planetary, passing through families, regions, states”. A health crisis that simultaneously intensified a humanitarian crisis and revealed the dearth of public policies geared towards social and health issues, further exacerbating the already poor living conditions of vulnerable populations. At the same time, it also triggered a movement for the transformation of health practices based on discussions of the challenges of training in public health during the pandemic, especially in light of growing health inequalities.

## The issue of inequality in Brazil, with a focus on health

There is ample evidence in the literature of the global nature of the inequality afflicting the populations of the poorest countries in particular, a phenomenon whose intractability makes it one of the most serious problems facing administrators, professionals, and public policymakers in this area. This same literature has highlighted the impact that socioeconomic characteristics have on the health of populations and the fact that economic, social, cultural, health, material, and symbolic resources are distributed extremely unequally in most societies ( Deaton, 2013 ; Marmot, 2017 ). For Marmot (2017) , an environment in which there is inequality, especially in terms of access to education, is one where social integration is hard to achieve.^5^

In Brazil, the persistence of inequality is indicative of the historical and structural roots of this problem, which are also found in professional training in the area of health. The emergence of the new coronavirus offered an opportunity for us to rethink health training and discuss the role of public health under a health crisis. According to Toneto, Ribas, and Carvalho (2021), one of the cruelest faces of the pandemic was the fact that given the paucity of investments in social programs, the same socioeconomic inequalities that exacerbated its effects by raising the risk of contagion and the number of deaths from the virus could themselves be responsible for deepening the crisis, undermining the prospects for recovery for Brazil and leaving the country even more vulnerable to new crises.

According to World Bank data from 2017 reported in *Um ajuste justo: análise da eficiência e equidade do gasto público do Brasil* [A fair adjustment: an analysis of the efficiency and equity of public spending in Brazil], between 2003 and 2014, Brazil experienced a period of economic and social progress in which more than 29 million people were lifted out of poverty and inequality decreased significantly. (The Gini coefficient fell 6.6% in the same period, from 58.1 to 51.5.) The income level of the poorest 40% of the population increased by an average of 7.1% in real terms over this period, compared to the 4.4% income growth observed in the general population ( Banco Mundial, 2017 ). Between 2002 and 2015, there was a significant drop in income inequality “at levels and of a quality that had not been seen in Brazilian history” ( Campello et al., 2018 , p.55). In the following years, from 2014 to 2016, the country experienced an economic recession. The pandemic hit Brazil when it was still recovering from this and exposed the country to a major health challenge. According to IBGE data from 2020, the total number of poor people exceeded 51 million in 2019, of whom 38.1 million were black ( IBGE, 2020 ).

The number of covid-19 cases and deaths in Brazil clearly reflects the country’s deep racial and regional inequalities (Pires, Carvalho, Rawet, 2021). Measures to curb the spread of the virus, such as the use of hand sanitizer, mask-wearing, hand-washing, and even social distancing, affected different Brazilian realities in different ways, notably in situations where basic rights such as health, employment, and housing were absent. Indigenous, *quilombola* , and river-dependent people, homeless people, refugees, itinerant groups, people who live in favelas and peripheries, people with HIV/AIDS, people with no formal employment, and other groups all share the fate of being marginalized in Brazilian society, which makes them even most vulnerable when a crisis strikes.

It is worth highlighting the correlation between socioeconomic vulnerability and the severity of disease and death from covid-19: the intersection of factors like racism, sexism, and income differences led to increased rates of infection, hospitalization, and death in the country (Pires, Carvalho, Rawet, 2021). In São Paulo city, for example, lower incomes areas were the most affected. Most deaths occurred in public hospitals and among people of African, Asian, or indigenous descent; 19.1% of covid-19 deaths of people on an ICU waiting list occurred in public hospitals, but just 1% occurred in private hospitals ( Bermudi et al., 2021 ).^7^These data are significant in the context of increasing inequality and a dearth of public policies for education, health, and social protection. In this sense, what are the challenges of professional practices in the post-pandemic era?

## An inflexion in the debate: the role of education in social and cultural transformation

It is important to recognize changes in the higher education of health professionals that had been encouraged through public policies, with the approval of the National Program for the Reorientation of Professional Training in Health (Pro-Health) in 2005 and the National Policy for Continuing Education in 2009 (in addition to the More Doctors Program). These have driven initiatives to change the offer of training in the last two decades, transforming training processes with a focus on social relevance and socioeconomic and cultural considerations to provide care in communities in a way that takes account of their complexities ( Matias et al., 2019 ; Haddad, Cyrino, Batista, 2018). However, these same programs have been dismantled or downgraded in recent years as a result of political decisions to slash investments in education and public health.

The resulting scenario is something of a minefield, where there is much resistance to change and the education on offer is still often of a traditional nature, focusing on developing a particular discipline with its associated skillsets. This in turn reinforces outdated healthcare practices and impairs the quality of the services available to the population ( Peduzzi, 2009 ).

The methodologies used in health education are still largely content-based and fragmented, centered on diseases, individual clinical cases, and the accumulation of technical and scientific information. As a result, the profile of newly trained professionals does not match the profile required in the field. According to Loureiro (2008) , the changes health services have undergone mean old paradigms and traditional teaching methods need to be overhauled. Professionals must be able to identify which population groups are vulnerable or at risk or have had their rights violated so that, in the context of a health crisis, they can be more sensitive to such inequities and disadvantages.

For Morin (2005) , university education has developed in such a way as to separate objects from their contexts. Subject matter is divided into disciplines that are not integrated, which means the full complexity of the health reality is not addressed. As a consequence, such education lacks inventive, reflexive, and creative “substance.” In response, there is a clear need to rethink the university, making it relevant to society; to take quality academic outputs to society through actions in communities, with communities, and for communities, making them more effective as actors in the process, with rights and responsibilities.

This discussion is therefore of great importance, in that it views professional training as a field in which change is wrought to traditional educational paradigms, as a pedagogical act that brings health professionals closer to pedagogical practices constructed socially in concrete reality, which seek to establish an “intimacy”, as Freire would say, between curricular knowledge and social experience.

## Bringing Paulo Freire and Edgar Morin into dialogue with the knowledge needed for a socially oriented practice

This section discusses the training of health professionals in the face of demands arising from society on the basis of the following questions: How can the ideas proposed by Paulo Freire and Edgar Morin be harnessed in the training provided for today’s health professionals? And how can socially responsible training be propositional for the reduction of socioeconomic inequalities in the country?

These questions raise the following issue: since inequalities are historically entrenched in Brazilian society and are part of a perverse social fabric – and thus also in the field of health – the only way to reduce inequality and improve primary care and regionalized care networks is by adopting strategies to provide qualified training and appreciation of health professionals, the integration of knowledge and the whole diversity of experiences, and the creation of spaces that facilitate reflections across theory and practice – a task that the state, in all its facets, is largely responsible for undertaking.

As Sevalho (2018 , p.82) puts it in his essay “O conceito de vulnerabilidade e a educação em saúde fundamentada em Paulo Freire” [The concept of vulnerability and health education based on Paulo Freire], “as of 2012, we can state in the strongest terms that in the country that made Paulo Freire its Patron of Education, his ideas are largely unknown and undermined by those who defend the subordination of the population to the interests of economic elites and systematically marginalized by official institutions and policies”.

The effectiveness and equity of health systems need to be improved, as does the training provided for their workers, rooted in a sense of social responsibility and a quest to find answers to the challenges inherent to meeting the population’s health care needs. For the education process to take this shape, competencies must be built in dealing with vulnerable populations and a perception developed that socially oriented training will help health systems provide equitable access to services. According to Paim et al. (2011) , it is essential to address the root causes of health problems to help shape more comprehensive care models, despite the numerous difficulties. Building a healthcare model with a national footprint is a complex process that must take account of multiple factors related to the social determinants of health.

It should be noted that the National Policy for the Promotion of Health provides recommendations for education, training, and capacity-building for primary care workers designed to enhance the commitment and critical and reflective capacity of health workers and administrators vis-a-vis different social realities, extracting from these realities the essence of a community practice that is dialogic, dynamic, and socially responsible ( Brasil, 2010 ). In the words of Sperling (2018 , p.341), “primary care is not only the first level of structured contact for patient care; it is also undoubtedly a field of disputes over the production of signifiers and signifieds in the process of caring for human lives.” Health systems are propitious for the application of scientific and technological knowledge, and their innovations influence concepts and principles associated with the functioning of societies and the development of citizenship.

It is important to highlight that notwithstanding the differences between Paulo Freire’s and Edgar Morin’s proposals, both believed training should be underpinned by particular kinds of knowledge so that education could be built on the basis of community practice and concrete reality to train professionals with sensitivity to social issues. According to Brandão (2017 , p.97-98), Paulo Freire’s concept of education can be summed up in seven sentences: “1) Live your life; 2) create your destiny; 3) learn your thinking; 4) share your learning; 5) say your word; 6) transform your world; 7) write your story.”

In Paulo Freire, the knowledge needed for education to transform society should be developed critically with what could be called “epistemological curiosity.” It is a kind of knowledge that is built socially in community practice and concrete reality and aims to establish an “intimacy” between curricular knowledge, social experiences, and common-sense knowledge. In this sense, education, still according to Freire (2019, p.19-20), means adopting a mindset inspired by humanitarian ethics: “the scientific preparation of the teacher must be aligned with their ethical rectitude ... Scientific education, ethical stance, respect for others, coherence, ability to live and learn from difference ... As a conscious presence in the world, I cannot escape ethical responsibility in my movement in the world.”

Education should be marked by a deep interpretation of social problems and be open to revisions, keeping prejudices at bay when analyzing problems to avoid distortions and strengthen arguments through dialogue ( Freire, 1987 ). In other words, education must reject traditional, linear, reductionist, and simplistic thinking, which is incapable of providing answers that truly speak to the problems encountered in reality.

For Edgar Morin (2002 , p.13), “fundamental” knowledge concerns the seven “black holes” of education that are scattered throughout the teaching and learning process: (1) “the blindness of knowledge” – i.e., a tendency towards “error and illusion” in regard to reality; (2) “the principles of pertinent knowledge,” by which “increasingly multidisciplinary, transversal, multidimensional, transnational, global, and planetary” knowledge is coordinated and organized (p.36); (3) “teaching the human condition,” meaning the position of the individual in the world to which all sciences converge; (4) “teaching the earthly identity,” which covers the diversity of languages, cultures, and social organizations; (5) “confronting uncertainty” – the unexpected and unforeseen events of human history – or “unknown adventures,” despite their social, cultural, economic, and health-related determinants; (6) “teaching understanding” of the planetary condition, especially in the age of globalization, with its volume of information that cannot be processed and organized by people; and (7) “human genus, or anthropo-ethics,” which corresponds to social participation, autonomy, and social responsibility.

According to Morin (2002) , the existence of these “black holes” in education demonstrates the need to understand what he defines as “ecology of action”: the attitude that is taken when an action that is triggered extrapolates the intentions of its instigator and may even be construed in a way that contradicts the original intention. Still according to Morin, human history is full of examples of this nature. In this sense, decision-making should involve developing strategies that can be corrected during execution in response to unforeseen circumstances and new information.

Let us take a closer look at each of these types of knowledge Edgar Morin proposes are needed for education and see how they coincide with Paulo Freire’s thoughts on the subject.

(1) “The blindness of knowledge: error and illusion.” Here, Morin discusses the difficulties and tendencies to “error and illusion.” He writes that knowledge should make “known what knowing is,” meaning that education should embrace the cultural characteristics of human knowledge, in its contexts and complexities. “Knowledge cannot be considered a ready-made tool that can be used without examining its nature” (Morin, 2002, p.14).

In Freire’s view (1992), knowledge is constructed socially in concrete reality: “respect for cultural differences, respect for the context one enters, criticism of ‘cultural invasion,’ sectarianization, and advocacy of the radicalism I speak of in *Pedagogia do oprimido* [Pedagogy of the Oppressed]” (p.44). In this sense, knowledge (perceptions, translations, and reconstructions that individuals experience throughout life) built from reality involves taking a critical stance towards problems and can render transformations in societies.

(2) “The principles of pertinent knowledge.” This, according to Morin, is knowledge that is capable of assimilating global, partial, and local problems, of forging connections between the parts and the whole, and thereby calls fragmented knowledge into question. In the words of Morin (2002, p.41), “we should teach methods of grasping mutual relations and reciprocal influences between parts and the whole in a complex world.”

For Freire (1987 , p.55), dialogue begins when deciding on the content of educational curricula, which should be arranged on the basis of concrete, real-world situations “reflecting the combined aspirations of the people.” The objective and subjective dimensions of knowledge must be understood as concrete aspects of the reality in which the act of cognition occurs. It is this dialectical unity, Freire asserts, that generates coherence between “acting” and “thinking” about reality in order to transform it. Therefore, the ultimate goal of awareness-raising knowledge is to engage in culturally and socially transformative action ( Brandão, 2017 ).

(3) “Teaching the human condition.” Since man is at the same time physical, biological, psychic, cultural, social, and historical, Morin argues that cultural identity-building must be part of the teaching-learning process in the form of disciplines that interrelate to discuss complex identities, organize fragmented knowledge, and bring forth human diversity.

For Freire (2019) , teaching means recognizing and “assuming” a cultural identity. In other words, the social grouping must acknowledge the historical, social, cultural, and political experiences of individuals and communities. Man, in the words of Freire (1987 , p.7), “engages in the ambiguity of the human condition and the contradictions of the historical adventure, projecting himself in the continuous recreation of a world that simultaneously hinders and fosters the drive to overcome the limits of human consciousness.”

(4) “Teaching the earthly identity.” In the words of Morin (2002 , p.59), “a human being is a reasonable and unreasonable being who can be subdued and excessive. Subject to intense unstable affectivity, he smiles, laughs, and cries but is also able to understand objectively. He is serious and calculating but also nervous, anguished, playful, excitable, ecstatic; he is a being of violence and tenderness, love and hate; a being invaded by the imaginary who can recognize the real.” In this sense, education should work with the multifaceted, historical, intertwined, and inseparable destinies of individuals.

For Freire (1987 , p.24), human understanding is a result of “the mind becoming more conscious.” Critical understanding of reality is built through the action of individuals or groups. Neither objectivity nor subjectivity should be denied in the analysis of reality, provided this ongoing dialectic becomes the creation of consciousness.

(5) “Confronting uncertainties.” Here, Morin (2002 , p.86-87) draws attention to the great questions about what can be known, since knowledge is complex and stems from cultural, social, individual, collective inputs. He emphasizes the importance of “preparing our minds” for great, often unexpected occurrences and disasters. Human history is an uncertain adventure that does not evolve in a straight line, and the future remains unpredictable.

Freire takes a parallel route, since for him knowledge also comes from cultural, social, individual, and collective contexts, and ideals and proposals for social action emerge from actions in the world and, at the same time, from actions affecting the self. The times of geographic spaces and their cultural relations are drawn along complex and uncertain paths between convergences in and among cultures and convergences of different domains of creation of new ideas.

(6) “Teaching understanding.” Morin (2002) confronts uncertainty by taking ambiguity, contradiction, and unpredictability as constituent aspects of the world, valuing acts of teaching human understanding that encompass such characteristics. Understanding has become crucial for humans as a precondition for solidarity in exchanges of knowledge. Understanding means, in Morin’s terms, learning together, as it includes empathy, openness, kindness, and generosity. There are, he says, many obstacles to understanding. One that is often present, especially in academic circles, is egocentrism, which has the effect of impairing the teaching-learning process. For Morin (2002, p.97): “in fact, misunderstanding the self is a major source of misunderstanding the other. Our own shortcomings and weaknesses are masked, which makes us impervious to the shortcomings and weaknesses of others.” Understanding oneself and others is therefore a prerequisite if educational processes are to avoid being based on preconceived ideas, random assumptions, and the inability to self-criticize. This perception permeates the entirety of Paulo Freire’s work, since for him teaching is an act of reciprocity and acceptance (Freire, 2019).

(7) “Ethics for the human genus, or anthropo-ethics.” Morin (2002) takes the conscious decision to embrace the complexity of the human condition, uncertainty, and unpredictability as part and parcel of life. Anthropo-ethics means “respecting in others both difference from and sameness with oneself” (p.106).

For Freire (2019) , the ethical subject is permanently exposed to the transgression of ethics, but it is necessary to be guided by ethics without erring towards hypocritical moralism. The universal ethics of the human being is, for him, indispensable to human coexistence: “As a conscious presence in the world, I cannot escape ethical responsibility in my movements in the world” (p.20). Also according to Freire (1996 , p.99), “the hegemonic education immobilizes and hides truths,” it is a type of education that is used to create a passive, submissive consciousness with the purpose of taming the present to ensure the established and controlled order is propagated into the future.

## Post-pandemic scenarios and recommendations: training in health and recognition for health work

Let us look at some scenarios for post-pandemic health education based on the ideas of these two thinkers and in contexts marked by the socioeconomic inequalities already alluded to in this text. The connections between inequality and health can be perceived in the daily life of Brazilian society and its health services. In addition, the country is facing a public health catastrophe, which calls for a kind of training that takes account of the cultural characteristics of human knowledge in its contexts and in its complexities, or, as Paulo Freire (1992) put it, knowledge that is constructed socially in the concrete reality and that respects cultural differences and the historical complexities of societies.

The pandemic has highlighted the importance of care and access to health services for the sustainability of the living conditions of the most vulnerable populations. Just as several areas of knowledge will need to be adapted to the new living conditions, health services and health workers will also increasingly need to adapt the way health care is delivered, restructure services, and think up new ways of working that take account of prevailing inequalities, inequities, and injustices. This public health crisis requires a redoubled commitment to preparing for global public health, which includes integrating policies and strategies targeting the structural causes of inequalities, such as universal populational approaches and intersectoral governance to mitigate the damage caused by the pandemic (Nogueira, Rocha, Akerman, 2021).

As noted by Noronha et al. (2021) , a virus does not choose hosts based on status and income, but inequalities become evident as the virus spreads, resulting in inequities in access to health services and facilities, unequal capacity to adopt preventive measures, and differences according to pre-existing health conditions related to socioeconomic, educational, and environmental inequities. Although the pandemic affected people of all classes and categories, the intensity of this impact was generally different; inequities came forth. The developments and challenges that a pandemic imposes on each family and each person are profoundly unequal, especially when there are significant preexisting inequalities. In terms of health, this therefore means professional skills and competences must adapt to the changes in the practices, techniques, and scientific evidence they use, and above all in response to the social demands of the population. The social must be integrated into everyday practices and the production of care.

It could be argued, in this sense, that the pandemic represents an opportunity to transform the way health professionals work, equipping them to identify sources of information, formulate research questions, critically analyze evidence, develop research of societal relevance, and apply the results in professional practice; all of which is also related to economic, political, and cultural issues (Faria, Almeida-Filho, Oliveira-Lima, 2021). Therefore, “critical knowledge and practice” is a prerequisite for the training and development of professionals capable of adapting to daily life, social relations, and cultural diversities, as Paulo Freire (1987 , 1992 ) would say.

This debate is fundamental and can help us rethink training on the basis of different theoretical and methodological concepts designed to strengthen socio-educational attributes, curbing the discourse that sees biomedical truth an incontestable – a discourse that spills over into academic health training environments, producing certainties and truths that, with their curative/medicalizing nature, downplay the importance of knowledge produced in community practice, reflecting the living conditions of communities. Based on this reality, the social is embedded in the everyday production of care, making the training of health workers a social outcome and social determination the basis for understanding inequalities ( Almeida-Filho, 2011 ).

It is important to rethink training for health in connection with changes in undergraduate and graduate curricula designed, among other things, to make health services more integrated, focused on problem-solving, and sensitive to local realities. It is worth mentioning that many professionals feel powerless in the face of the sheer scale of the social dimension. That is why training must prioritize a model of care that is linked to social problems, the health needs of communities, and the upholding of the principles of SUS.

## Final considerations

The process of transforming professional practices in healthcare is reminiscent of the “crossing” with which Guimarães Rosa (2019) ends *Grande sertão: veredas* , originally published in 1956. In this crossing, in which education is transformed, it takes on a variety of meanings whose practical implications must consider ambiguity, contradiction, and unpredictability as inherent to the world. It must also consider the conscious decision to take the complexity of the human condition on board; to seek to understand oneself and others so that the pedagogical process does not reflect preconceived ideas, as Freire and Morin would say. In other words, we are talking about a form of education that is capable of assimilating global and local problems, calls into question fragmented knowledge, and encourages the development of inclusive citizenship.

The covid-19 pandemic brought into sharp relief the scale of the impacts that a health emergency can have on the health of populations and tested the effective response capacity of countries in Latin America and the Caribbean. The disease became one of the main causes of death in the region and exacerbated inequalities, as these countries were already “breeding grounds” of poverty, violence, and social injustice. Knowledge about how the health crisis affects societies and reveals social inequalities and what consequences and challenges arise when new inequalities take root is important for a better comprehension to be reached on how to tackle this context mindfully and in ways that are consistent with social demands.

How does social inequality in Latin America, especially Brazil, exacerbated by the pandemic, impact or challenge social protection measures and education and training for health workers? For Brazil, as in the whole region, priority must be given to resuming social protection measures and investments in health training at this crucial moment in history when Brazilian society is handicapped by inequality of so many kinds. For health professionals, learning in new formats will be key, with a sharp ear and eye to the complexity of the reality they face. To overcome the crisis, we must rethink our educational models in the field of health, taking inspiration from the ideas of Paulo Freire and Edgar Morin, so that they enable dispersed knowledge to be organized and highlight the diversities and complexities of human beings.

For Freire and Morin, understanding involves empathy, identification, generosity, and solidarity. Henceforth, social solidarity should be the guiding light for health education based on the understanding that education is a social practice. These new realities are challenging and require health professionals to be able to find new solutions in response to respond to a wide range of problems facing the population, encourage mutual encounters, expand the frontiers of knowledge, propose social protection policies, see the “invisible thing, [the] absent thing,” and understand the unpredictable and the uncertain, as Calvino (1990 , p.59, 66) reminds us, through permanent dialogue with all forms of knowledge, especially popular knowledge.

## NOTES

1 A study published in *The Lancet* on March 10, 2022, made an important contribution to understanding the impact of covid-19 on mortality rates in 191 countries. The authors’ estimates suggest that the impact of mortality was more devastating than had been documented in official reports. According to the study data, which were gathered until December 31, 2021, official statistics recognized 5.94 million deaths from the disease, but the number estimated in the study is three times higher (18.2 million) ( Wang et al., 2022 ).

2 Programa Andifes de Mobilidade Virtual Internacional: Destino Brasil [ANDIFES Program for Internacional Virtual Mobility: Destination Brazil].

3 In the text, the expression “care economy” indicates social, economic, cultural, and health factors, especially gender issues, which positively influence the living conditions of populations and reduce risk factors, especially among more vulnerable populations, because of social protection policies and investments in strategic areas, such as health and, within it, professional training. It encompasses socially relevant issues that connect to theoretical and practical meanings about the provision of care in a way that contributes to a quality life with equality, as Paulo Freire would say.

4 All the data related to the pandemic, inequality, and public policies in Latin America and the Caribbean can be found in the material for the international course “La pandemia y la salud pública en Latinoamérica.”

5 It is important to distinguish between health inequalities and health inequities. Health inequalities refer to differences in health status related to differences in access to services and care. Health inequities refer to inequalities that could be avoided and are therefore unfair (Kawachi, Subramanian, Almeida-Filho, 2002; Barreto, 2017 ).
